# In-vitro evaluation of retention in cement-retained implant prostheses with and without screw access channel

**DOI:** 10.6026/973206300212608

**Published:** 2025-08-31

**Authors:** Vivek V., Aishwarya Mahajan, Sonal Srivastava, Darakshan Saifi, Pinki Bankoti, Chandana Nair

**Affiliations:** 1 Department of Dental Surgery, VMMC & Safdarjung Hospital, New Delhi, India; 2 Department of Prosthodontics and Crown and Bridge, Institute of Dental Science, Bareilly, Uttar Pradesh, India; 3 Private Practitioner, Smile and Braces Multi-Speciality Dental clinic, Custom office lane, Near Prabhat khabar office, Kargil chowk, Sitamarhi, Bihar, India

**Keywords:** Cement-retained implant prostheses, glass ionomer cement, masticatory loads

## Abstract

The retention of cement-retain implants prostheses with and without screw-access channels, using Glass Ionomer Cement (GIC) and resin cement. The methodology involved meticulous specimen mounting, scanning of abutments, 3D printing of resin patterns, casting, recovery, finishing, cementation, and tensile testing. Cement-retained implant restorations without access holes are recommended in cases with high aesthetic demand and increased masticatory load. Incorporating access holes aids in retrievability, retention, and occlusion, and decreases the extrusion of cement in the peri-implant space, but may compromise aesthetics. Thus, cementation of the implant prosthesis is recommended in cases with increased masticatory loads.

## Background:

In recent years, implant dentistry has advanced significantly, offering a remarkable solution to restore natural appearance, speech, aesthetics, overall health, and functionality for patients [1]. This progress has led to widespread acceptance of implant-supported prostheses due to their outstanding results. Two standard methods for retaining implant-supported superstructures are screw-retained and cement-retained implant restorations [2]. Screw-retained restorations are preferred for their effective complication management [3]. However, minor errors during fabrication can lead to discrepancies between the prosthesis and implants, which can impact the fit. In contrast, cement-retained restorations are easier to create using conventional methods and are more cost-effective. They allow for adjustment of minor size differences and angulations, providing a superior fit [4]. However, maintaining and retrieving cement-retained crowns can be challenging and excess cement can lead to peri-implant diseases [2]. To address challenges with retrievability and excess cement, some propose incorporating access holes in cement-retained restorations [5]. However, there is debate over their use due to aesthetic concerns [4]. A technique combining elements of screw-retained and cement-retained restorations has been proposed to address both concerns effectively [6]. Various factors influence retention strength of dental cement in the oral environment during mastication [7]. Accurately replicating these variables in research studies is crucial for clinically relevant conclusions. A comparative analysis of the tensile strength between cement-retained prostheses with and without access holes is essential to support clinical advocacy effectively [8]. This analysis would provide valuable insights into mechanical performance and durability, aiding clinicians in decision-making [9]. Therefore, it is of interest to compare and evaluate retention in cement-retained implant prostheses with a screw access channel versus those without a screw access channel.

## Study designs:

## Mounting of specimen:

The initial step of the experiment involved mounting the specimens into clear acrylic resin, ensuring accurate positioning and stability. For this purpose, a customized square metal framework that could be split into two halves was fabricated, serving as the foundation for mounting the specimens. Thirty-six titanium abutments (Sigdent dental implants, Israel) were screwed onto 36 dummy implants (Sigdent dental implants, Israel), using a manual torque wrench (Sigdent dental implants, Israel) to tighten the abutments to a standardized torque of 35 Ncm, ensuring consistency across all specimens. The access holes were filled with additional silicone putty body material (Aveu, Korea). The horizontal arm of the surveyor (Marathon, Korea) was then extended from the top of the vertical arm to facilitate the precise positioning of the implant abutment assembly attached to the surveying mandrel in the middle of the square metal frame. Then the framework was poured with clear acrylic resin to form a specimen block.

## Scanning of abutments using a lab scanner:

Before scanning, the clear resin blocks with abutment-implant assemblies were sprayed with Easy Spray (Alphadent, Germany) to prepare the surface for scanning. The clear resin blocks containing the abutment-implant assemblies were then placed in the extraoral lab scanner (Medit T310, Korea), and high-resolution scans were performed to capture detailed digital images of the specimens.

## 3D printing of resin pattern:

The digital scans obtained were used to design resin patterns for the fabrication of metal copings. Two types of resin patterns were designed: metal crowns with access holes and metal crowns without access holes. A design for metal loops was created and incorporated into the resin pattern design. Once the resin pattern designs were finalized, they were 3D printed using a high-precision 3D printer (ELGOO, China).

## Casting of resin pattern:

The resin patterns were sprued (Bego, Germany) using a 2.5mm sprue at the centre of the loop and were placed on a crucible former (Unident, India) at a 45-degree angle. The investment material (Bego, Germany) was mixed in the proper ratio and poured into a casting ring (Bego, Germany) lined with a cellulose casting ring liner (GC Dental, Japan). The casting ring assembly was then placed in a microprocess-controlled unit for burnout. The casting process was carried out using an induction casting machine, using Ni-Cr metal pellets (Bego, Germany).

## Recovery and finishing of casting:

The castings were retrieved by tapping the casting ring with a mallet, and the sprues were cut. Any remaining investment material was then removed using a sandblasting procedure. The copings were examined for defects and then finished and polished using metal finishing stones, burs, and sandblasting (Unident, India) to achieve copings with smooth surfaces and proper margins.

## Cementation procedure:

Two types of cement were used for cementation: glass ionomer cement (GIC, ShofuDental, Japan) and resin cement (SAC, Calibra Universal, Dentsply Sirona, India). GIC was manipulated and then evenly spread over the intaglio surface of the copings. For cementation using resin cement, the copings were pretreated with sandblasting, a silane coupling agent (Monobond-N) was applied, and the resin cement was dispensed onto the copings and evenly spread with a probe.

## Group description:

The specimens were divided into two groups, A and B, based on the fabrication technique. These groups were further subdivided into subgroups based on coping design and cement type.

Group A: Metal coping fabricated without an access hole.

[1] A1: Metal coping without access holes luted with GIC.

[2] A2: Metal coping without access holes luted with Self Adhesive Resin Cement.

Group B: Metal coping fabricated with an access hole.

[1] B1: Metal coping with an access hole luted with GIC.

[2] B2: Metal coping with access hole luted with Self Adhesive Resin Cement.

## Tensile testing:

The tensile testing procedure involves evaluating the retention strength of the copings using a Universal Testing Machine (UTM). Before testing, the copings were subjected to compressive cyclic loading and then stored in artificial saliva for 24 hours to simulate oral conditions. The copings were then fixed onto the UTM, and tensile force was applied along the long axis of the specimen until dislodgement occurred. The force required to dislodge the copings was recorded in Newtons. Data was presented in Mean and standard deviation. A student's Independent T-test was performed to determine if there was a significant difference in variables between the two groups.

## Results:

The tensile strengths were as follows: A1 - 775.4, A2 - 1218.8, B1 - 724.7, and B2 - 1175.0, revealing that the copings with access hole cemented using GIC had the lowest tensile strength. In contrast, the teeth dealt with without an access hole, cemented using resin cement, had the highest tensile strength (Table 1 - see PDF). The tensile strength of cement-retained implant restorations cemented with GIC remains the same regardless of whether they have an access hole or not. This difference is statistically insignificant, with p-values of 0.115 (Table 2 - see PDF). The tensile strength of cement-retained implants restorations, whether they have an access hole or not, remains the same when cemented with resin cement. This difference is statistically insignificant, with a p-value of 0.172 (Table 2 - see PDF). Cement-retained implant restorations, whether with or without an access hole and cemented with resin cement, demonstrate better tensile strength than those cemented with GIC. This is supported by a p-value of 0.001, which is highly significant (Table 2 - see PDF).

## Discussion:

In dentistry, implantable materials play a crucial role in ensuring the success and longevity of dental implants. Titanium and its alloys, cobalt chromium alloys, austenitic Fe-Cr-Ni-Mo steels, tantalum, niobium and zirconium alloys, precious metals, ceramics, and polymeric materials are among the main categories of implantable materials utilized. Titanium, particularly Titanium-6 Aluminum-4 Vanadium (Ti6Al4V), is widely favored due to its biocompatibility and ability to passivate upon contact with normal tissue fluids, thereby minimizing biocorrosion [10]. Retention is paramount for the longevity of implant prosthesis, influenced by factors such as taper, height, surface treatment, and fitting of prosthesis components. Machined abutments with a 6° taper are commonly used, but a 00 taper ideally provides the best retention [11]. The minimum abutment height required for cement-retained restorations with predictable retention is 5 mm [12]. Surface roughening techniques, such as diamond bur roughening or grit blasting, enhance the mechanical retention of cements. CAD-CAM 3D-printed crowns offer a superior marginal fit and adaptation, with sandblasting further enhancing their surface for cementation [11]. Various cement types, including glass ionomer cement (GIC) and resin cement, are used in clinical practice for cementing fixed partial prostheses due to their high retention levels. Resinous cements offer superior retention but may cause soft tissue toxicity when extruded into the peri-implant space. To mitigate this, occlusal access holes are incorporated, reducing the risk of cement extrusion [13]. Screw loosening can occur in both cement-retained implant restorations (CRIR) and screw-retained implant restorations (SRIR), with CRIR potentially offering better longevity due to the cement space compensating for axial forces. Incorporating screw access holes facilitates restoration, removal, and replacement in case of failures [14, 15]. The presence of screw access channels or mini access holes affects crown structure and aesthetics, but they are essential for facilitating restoration retrieval. Various methods exist for retrieval, including the use of provisional cement, set screws, or guide holes, each with its advantages and limitations [16]. Retrievable, cement and screw-retained crowns (CSRC) offer the benefits of both CRIR and SRIR but may present issues with ceramic fracture. Standardized guidelines for the optimal diameter of screw access channels are lacking, and excessive preparation can lead to compromised aesthetics and restoration strength [17, 18-19]. Tensile testing is used to establish operational load limits for metals and alloys. The tensile test measures a material's ability to withstand stress (force per unit area). The response of a tensile sample to the application of an increasing stress can be described in terms of elastic and plastic behaviour [20]. Tensile force experiments demonstrate that the type of cement significantly influences retention, with resin cement exhibiting higher retention values compared to other types. CRIR cemented with resin cement demonstrates superior tensile strength compared to those cemented with GIC, further highlighting the importance of cement selection in ensuring restoration longevity [21]. Da Rocha *et al.* (2013) [21] studied the influence of screw access on the retention of cement-retained implant prostheses. They concluded that the screw access channel did not significantly diminish the crown's retention capability. Alvarez-Arena *et al.* (2016) [8] studied the retention strength of luting agents and concluded that the resin composite & Resin Modified Glass Ionomer Cement have the highest retentive strength. Overall, meticulous consideration of material selection, cement type, and restoration design is crucial for the success of implant prosthesis, ensuring optimal retention, aesthetics, and longevity.

## Conclusion:

The copings cemented with resin cement have better tensile strength compared to those cemented with GIC, so the use of resin cement is recommended. Additionally, resin cement also acts to absorb shock, thereby reducing screw loosening in crowns that bear increased masticatory loads. Thus, the incorporation of an access hole in the design of the prosthesis depends on the patient's requirement and the clinician's judgement.
